# 2,4-Bis(3-chloro­phen­yl)-3-aza­bicyclo­[3.3.1]nonan-9-one

**DOI:** 10.1107/S1600536809009945

**Published:** 2009-03-25

**Authors:** P. Parthiban, V. Ramkumar, H. D. Santan, Jong Tae Kim, Yeon Tae Jeong

**Affiliations:** a Division of Image Science and Information Engineering, Pukyong National University, Busan 608 739, Republic of Korea; b Department of Chemistry, IIT Madras, Chennai, TamilNadu, India

## Abstract

In the mol­ecular structure of the title compound, C_20_H_19_Cl_2_NO, the bicyclic system adopts a twin-chair conformation with equatorial orientations of both substituents. The dihedral angle between the aromatic rings is 43.60 (2)° with respect to each other. The crystal structure is stabilized by weak N—H⋯O and strong C—H⋯O inter­actions.

## Related literature

For the biological significance, synthesis and stereochemistry of 3-aza­bicyclo­nonan-9-ones, see: Jeyaraman & Avila (1981 ▶). For similiar structures, see: Parthiban *et al.* (2008*a* ▶,*b* ▶,*c* ▶,*d* ▶,*e* ▶). For puckering parameters, see: Web & Becker (1967 ▶); Cremer & Pople (1975 ▶).
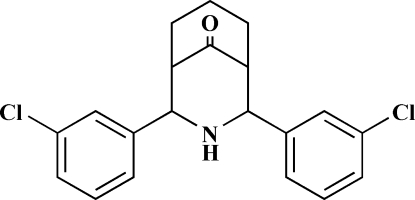


## Experimental

### 

#### Crystal data

C_20_H_19_Cl_2_NO
*M* *_r_* = 360.26
Orthorhombic, 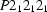

*a* = 6.9950 (14) Å
*b* = 12.180 (2) Å
*c* = 20.770 (4) Å
*V* = 1769.6 (6) Å^3^
*Z* = 4
Mo *K*α radiation
μ = 0.37 mm^−1^
*T* = 298 K
0.31 × 0.25 × 0.22 mm

#### Data collection

Bruker APEXII CCD area-detector diffractometer
Absorption correction: multi-scan (*SADABS*; Bruker, 1999 ▶) *T*_min_ = 0.875, *T*_max_ = 0.922
23614 measured reflections
4284 independent reflections
2762 reflections with *I* > 2σ(*I*)
*R*_int_ = 0.046

#### Refinement

*R*[*F* ^2^ > 2σ(*F* ^2^)] = 0.041
*wR*(*F* ^2^) = 0.084
*S* = 1.01
4284 reflections
221 parameters
H atoms treated by a mixture of independent and constrained refinement
Δρ_max_ = 0.16 e Å^−3^
Δρ_min_ = −0.25 e Å^−3^
Absolute structure: Flack (1983 ▶), 1756 Friedel pairs
Flack parameter: −0.05 (5)

### 

Data collection: *APEX2* (Bruker, 2004 ▶); cell refinement: *SAINT-Plus* (Bruker, 2004 ▶); data reduction: *SAINT-Plus*; program(s) used to solve structure: *SHELXS97* (Sheldrick, 2008 ▶); program(s) used to refine structure: *SHELXL97* (Sheldrick, 2008 ▶); molecular graphics: *ORTEP-3* (Farrugia, 1997 ▶); software used to prepare material for publication: *SHELXL97*.

## Supplementary Material

Crystal structure: contains datablocks global, I. DOI: 10.1107/S1600536809009945/gw2060sup1.cif

Structure factors: contains datablocks I. DOI: 10.1107/S1600536809009945/gw2060Isup2.hkl

Additional supplementary materials:  crystallographic information; 3D view; checkCIF report

## Figures and Tables

**Table 1:** Hydrogen-bond geometry (Å, °)

*D*—H⋯*A*	*D*—H	H⋯*A*	*D*⋯*A*	*D*—H⋯*A*
N1—H1*A*⋯O1^i^	0.83 (2)	2.35 (2)	3.129 (3)	155.4 (18)
C7—H7⋯O1^ii^	0.98	2.44	3.296 (2)	146

Symmetry codes: (i) 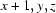; (ii) 
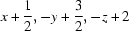
.

**Fig. 1.**
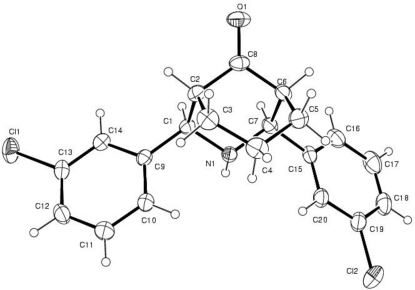
ORTEP of the molecule with atoms represented as 30% probability ellipsoids.

**Fig. 2.**
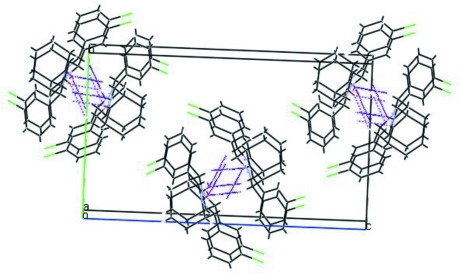
Packing diagram of molecules showing the N—H···O and C—H···O interactions.

## supplementary crystallographic information

### Comment

Due to their biological significance (Jeyaraman & Avila, 1981), the synthesis and stereochemistry of 3-azabicyclononan-9-ones are more important in current affairs (Parthiban *et al.*, 2008*a*,*b*,*c*,*d*,*e*). The title compound C_20_ H_19_ Cl_2_ N O, exists in twin-chair conformation with equatorial orientations of the *meta* chlorophenyl groups on both sides of the secondary amino group with the torsion angles of C8—C2—C1—C9 and C8—C6—C7—C15 are -177.78 (4)and 177.42 (3)°, respectively. A study of torsion angles, asymmetry parameters and least-squares plane calculation shows that the piperidine ring adopts near ideal chair conformation with the deviation of ring atoms N1 and C8 from the C1/C2/C6/C7 plane by -0.669 (2) and 0.704 (3) Å, respectively, Q_T_ = 0.617 (2) Å, q(2)=0.021 (2) and q(3)=0.617 (2) Å, θ = 2.71 (19)°. (Cremer & Pople, 1975; Web & Becker, 1967) whereas the cyclohexane ring deviate from the ideal chair conformation; the cyclohexane atoms C4 and C8 deviate from the C2/C3/C5/C6 plane by -0.545 (4) and 0.714 (3)°, respectively, Q_T_ = 0.562 (2) Å, q(2)=0.128 (2) and q(3)=0.549 (2) Å, θ = 12.7 (2)°. (Cremer & Pople, 1975). The aryl groups are oriented at an angle of 43.60 (2)° to each other.

The crystal structure is stabilized by weak N—H···O (3.129 (3)Å and strong C—H···O interactions [C1—H···O1 (3.46 (3)Å and C7—H···O1 3.296 (2) Å]. Interestingly,the same acceptor O1 is involved in trifurcated hydrogen bond with N1,C1 and C7 where the Oxygen atoms is at the apex forming a tripyramidal.

### Experimental

A mixture of cyclohexanone (0.05 mol) and *meta* chlorobenzaldehyde (0.1 mol) was added to a warm solution of ammonium acetate (0.075 mol) in 50 ml of absolute ethanol. The mixture was gently warmed on a hot plate till the yellow color was formed during the mixing of the reactants and cooled to room temperature. Then 50 ml of ether was added and allowed to stir over night at room temperature. At the end, the crude azabicyclic ketone was separated by filtration and washed with 1:5 ethanol-ether mixture till the solid became colourless. Recrystallization of the compound from ethanol gave X-ray diffraction quality crystals of 2,4-bis(3-chlorophenyl)-3-azabicyclo[3.3.1]nonan-9-one.

### Refinement

Nitrogen H atoms were located in a difference Fourier map and refined isotropically. Other hydrogen atoms were fixed geometrically and allowed to ride on the parent carbon atoms,with aromatic C—H =0.93 Å, aliphatic C—H = 0.98Å and methylen C—H = 0.97 Å. The displacement parameters were set for phenyl,methylen and aliphatic H atoms at *U*_iso_(H) = 1.2*U*_eq_(C).

### Crystal data

**Table:**

C_20_H_19_Cl_2_NO	*F*(000) = 752
*M**_r_* = 360.26	*D*_x_ = 1.352 Mg m^−^^3^
Orthorhombic, *P*2_1_2_1_2_1_	Mo *K*α radiation, λ = 0.71073 Å
Hall symbol: P 2ac 2ab	Cell parameters from 5421 reflections
*a* = 6.9950 (14) Å	θ = 2.6–22.5°
*b* = 12.180 (2) Å	µ = 0.37 mm^−^^1^
*c* = 20.770 (4) Å	*T* = 298 K
*V* = 1769.6 (6) Å^3^	Block, colourless
*Z* = 4	0.31 × 0.25 × 0.22 mm

### Data collection

**Table:**

Bruker APEXII CCD area-detector diffractometer	4284 independent reflections
Radiation source: fine-focus sealed tube	2762 reflections with *I* > 2σ(*I*)
graphite	*R*_int_ = 0.046
ω scans	θ_max_ = 28.3°, θ_min_ = 2.6°
Absorption correction: multi-scan (*SADABS*; Bruker, 1999)	*h* = −8→9
*T*_min_ = 0.875, *T*_max_ = 0.922	*k* = −16→16
23614 measured reflections	*l* = −27→16

### Refinement

**Table:**

Refinement on *F*^2^	Secondary atom site location: difference Fourier map
Least-squares matrix: full	Hydrogen site location: inferred from neighbouring sites
*R*[*F*^2^ > 2σ(*F*^2^)] = 0.041	H atoms treated by a mixture of independent and constrained refinement
*wR*(*F*^2^) = 0.084	*w* = 1/[σ^2^(*F*_o_^2^) + (0.0341*P*)^2^ + 0.181*P*] where *P* = (*F*_o_^2^ + 2*F*_c_^2^)/3
*S* = 1.01	(Δ/σ)_max_ = 0.002
4284 reflections	Δρ_max_ = 0.16 e Å^−^^3^
221 parameters	Δρ_min_ = −0.25 e Å^−^^3^
0 restraints	Absolute structure: Flack (1983), 1756 Friedel pairs
Primary atom site location: structure-invariant direct methods	Flack parameter: −0.05 (5)

### Special details

**Table:**

Geometry. All e.s.d.'s (except the e.s.d. in the dihedral angle between two l.s. planes)are estimated using the full covariance matrix. The cell e.s.d.'s are takeninto account individually in the estimation of e.s.d.'s in distances, anglesand torsion angles; correlations between e.s.d.'s in cell parameters are onlyused when they are defined by crystal symmetry. An approximate (isotropic)treatment of cell e.s.d.'s is used for estimating e.s.d.'s involving l.s. planes.
Refinement. Refinement of *F*^2^ against ALL reflections. The weighted *R*-factor *wR* andgoodness of fit *S* are based on *F*^2^, conventional *R*-factors *R* are basedon *F*, with *F* set to zero for negative *F*^2^. The threshold expression of*F*^2^ > σ(*F*^2^) is used only for calculating *R*-factors(gt) *etc*. and isnot relevant to the choice of reflections for refinement. *R*-factors basedon *F*^2^ are statistically about twice as large as those based on *F*, and *R*-factors based on ALL data will be even larger.

### Fractional atomic coordinates and isotropic or equivalent isotropic displacement parameters (Å^2^)

**Table:**

	*x*	*y*	*z*	*U*_iso_*/*U*_eq_	
C1	0.1155 (3)	0.91094 (15)	0.96470 (9)	0.0351 (5)	
H1	0.0839	0.8690	1.0035	0.042*	
C2	−0.0754 (3)	0.94712 (17)	0.93253 (10)	0.0425 (5)	
H2	−0.1483	0.9910	0.9635	0.051*	
C3	−0.0574 (3)	1.01301 (18)	0.86920 (11)	0.0522 (6)	
H3A	−0.1829	1.0398	0.8571	0.063*	
H3B	0.0236	1.0763	0.8768	0.063*	
C4	0.0248 (4)	0.94740 (18)	0.81344 (10)	0.0517 (6)	
H4A	0.0035	0.9874	0.7737	0.062*	
H4B	0.1617	0.9400	0.8193	0.062*	
C5	−0.0633 (3)	0.83391 (19)	0.80751 (10)	0.0493 (6)	
H5A	0.0141	0.7906	0.7782	0.059*	
H5B	−0.1894	0.8411	0.7886	0.059*	
C6	−0.0815 (3)	0.77124 (16)	0.87165 (10)	0.0405 (5)	
H6	−0.1583	0.7050	0.8646	0.049*	
C7	0.1088 (3)	0.73829 (16)	0.90524 (9)	0.0373 (5)	
H7	0.0767	0.7017	0.9459	0.045*	
C8	−0.1852 (3)	0.84501 (17)	0.91799 (9)	0.0417 (5)	
C9	0.2390 (3)	1.00628 (15)	0.98456 (9)	0.0353 (5)	
C10	0.3661 (3)	1.05578 (17)	0.94271 (10)	0.0485 (6)	
H10	0.3780	1.0292	0.9009	0.058*	
C11	0.4763 (4)	1.14476 (18)	0.96219 (12)	0.0589 (7)	
H11	0.5609	1.1773	0.9334	0.071*	
C12	0.4608 (3)	1.18510 (18)	1.02405 (11)	0.0514 (6)	
H12	0.5330	1.2452	1.0372	0.062*	
C13	0.3367 (3)	1.13481 (15)	1.06568 (10)	0.0430 (5)	
C14	0.2249 (3)	1.04678 (16)	1.04695 (9)	0.0399 (5)	
H14	0.1404	1.0148	1.0760	0.048*	
C15	0.2255 (3)	0.65933 (15)	0.86531 (10)	0.0388 (5)	
C16	0.2048 (3)	0.54727 (17)	0.87560 (12)	0.0543 (6)	
H16	0.1230	0.5222	0.9077	0.065*	
C17	0.3056 (4)	0.47232 (18)	0.83825 (15)	0.0679 (8)	
H17	0.2895	0.3975	0.8453	0.081*	
C18	0.4283 (4)	0.5073 (2)	0.79115 (13)	0.0619 (7)	
H18	0.4961	0.4570	0.7664	0.074*	
C19	0.4494 (3)	0.61781 (19)	0.78124 (12)	0.0514 (6)	
C20	0.3510 (3)	0.69381 (17)	0.81787 (10)	0.0457 (5)	
H20	0.3692	0.7684	0.8106	0.055*	
Cl1	0.32230 (11)	1.18133 (5)	1.14500 (3)	0.0681 (2)	
Cl2	0.60281 (11)	0.66335 (6)	0.72080 (3)	0.0794 (2)	
N1	0.2172 (3)	0.83753 (13)	0.92051 (8)	0.0358 (4)	
O1	−0.3405 (2)	0.82264 (14)	0.94107 (7)	0.0583 (4)	
H1A	0.324 (3)	0.8184 (15)	0.9342 (9)	0.033 (6)*	

### Atomic displacement parameters (Å^2^)

**Table:**

	*U*^11^	*U*^22^	*U*^33^	*U*^12^	*U*^13^	*U*^23^
C1	0.0369 (12)	0.0377 (11)	0.0307 (9)	−0.0016 (9)	0.0059 (9)	−0.0005 (9)
C2	0.0360 (12)	0.0472 (12)	0.0443 (12)	0.0048 (10)	0.0048 (10)	−0.0061 (10)
C3	0.0502 (15)	0.0476 (13)	0.0587 (15)	0.0068 (11)	−0.0079 (12)	0.0076 (11)
C4	0.0549 (14)	0.0579 (14)	0.0421 (12)	−0.0040 (12)	−0.0058 (11)	0.0153 (11)
C5	0.0462 (14)	0.0647 (14)	0.0370 (11)	0.0005 (12)	−0.0065 (10)	0.0008 (11)
C6	0.0358 (12)	0.0435 (12)	0.0421 (12)	−0.0079 (10)	−0.0046 (10)	−0.0016 (9)
C7	0.0386 (12)	0.0371 (11)	0.0363 (11)	−0.0059 (10)	−0.0013 (9)	0.0008 (9)
C8	0.0307 (12)	0.0577 (13)	0.0366 (11)	0.0013 (11)	−0.0007 (10)	0.0090 (10)
C9	0.0347 (11)	0.0353 (11)	0.0359 (11)	0.0048 (9)	0.0008 (9)	−0.0016 (9)
C10	0.0553 (14)	0.0469 (12)	0.0433 (12)	−0.0088 (12)	0.0088 (11)	−0.0102 (10)
C11	0.0656 (16)	0.0530 (15)	0.0580 (14)	−0.0152 (13)	0.0154 (13)	−0.0035 (12)
C12	0.0536 (15)	0.0388 (12)	0.0619 (15)	−0.0075 (12)	−0.0048 (12)	−0.0081 (11)
C13	0.0500 (13)	0.0381 (11)	0.0409 (11)	0.0058 (10)	−0.0065 (11)	−0.0082 (9)
C14	0.0406 (12)	0.0422 (11)	0.0368 (11)	0.0054 (10)	0.0017 (9)	0.0006 (9)
C15	0.0396 (12)	0.0355 (11)	0.0412 (11)	−0.0008 (9)	−0.0085 (10)	−0.0074 (9)
C16	0.0499 (15)	0.0423 (13)	0.0708 (16)	−0.0074 (12)	−0.0046 (12)	−0.0040 (11)
C17	0.0720 (19)	0.0362 (13)	0.096 (2)	0.0028 (13)	−0.0140 (17)	−0.0134 (13)
C18	0.0565 (16)	0.0550 (16)	0.0743 (18)	0.0137 (13)	−0.0127 (15)	−0.0296 (14)
C19	0.0460 (14)	0.0588 (15)	0.0494 (13)	0.0060 (11)	−0.0049 (11)	−0.0193 (11)
C20	0.0507 (13)	0.0386 (11)	0.0477 (12)	0.0012 (11)	−0.0006 (11)	−0.0109 (10)
Cl1	0.0959 (5)	0.0649 (4)	0.0435 (3)	−0.0048 (4)	−0.0084 (3)	−0.0171 (3)
Cl2	0.0814 (5)	0.0891 (5)	0.0675 (4)	0.0069 (4)	0.0235 (4)	−0.0261 (4)
N1	0.0305 (10)	0.0393 (9)	0.0376 (9)	0.0018 (9)	−0.0019 (8)	−0.0052 (8)
O1	0.0363 (9)	0.0793 (11)	0.0593 (10)	−0.0063 (9)	0.0087 (8)	0.0071 (9)

### Geometric parameters (Å, °)

**Table:**

C1—N1	1.465 (2)	C9—C10	1.382 (3)
C1—C9	1.505 (3)	C9—C14	1.390 (3)
C1—C2	1.557 (3)	C10—C11	1.390 (3)
C1—H1	0.9800	C10—H10	0.9300
C2—C8	1.493 (3)	C11—C12	1.380 (3)
C2—C3	1.546 (3)	C11—H11	0.9300
C2—H2	0.9800	C12—C13	1.369 (3)
C3—C4	1.520 (3)	C12—H12	0.9300
C3—H3A	0.9700	C13—C14	1.383 (3)
C3—H3B	0.9700	C13—Cl1	1.745 (2)
C4—C5	1.518 (3)	C14—H14	0.9300
C4—H4A	0.9700	C15—C16	1.389 (3)
C4—H4B	0.9700	C15—C20	1.385 (3)
C5—C6	1.541 (3)	C16—C17	1.390 (3)
C5—H5A	0.9700	C16—H16	0.9300
C5—H5B	0.9700	C17—C18	1.370 (4)
C6—C8	1.503 (3)	C17—H17	0.9300
C6—C7	1.556 (3)	C18—C19	1.369 (3)
C6—H6	0.9800	C18—H18	0.9300
C7—N1	1.462 (3)	C19—C20	1.382 (3)
C7—C15	1.510 (3)	C19—Cl2	1.742 (3)
C7—H7	0.9800	C20—H20	0.9300
C8—O1	1.218 (2)	N1—H1A	0.83 (2)
			
N1—C1—C9	111.35 (16)	O1—C8—C2	124.5 (2)
N1—C1—C2	108.66 (16)	O1—C8—C6	123.3 (2)
C9—C1—C2	113.05 (16)	C2—C8—C6	112.28 (17)
N1—C1—H1	107.9	C10—C9—C14	118.52 (19)
C9—C1—H1	107.9	C10—C9—C1	122.25 (18)
C2—C1—H1	107.9	C14—C9—C1	119.23 (18)
C8—C2—C3	107.59 (18)	C9—C10—C11	120.9 (2)
C8—C2—C1	107.01 (16)	C9—C10—H10	119.5
C3—C2—C1	116.25 (17)	C11—C10—H10	119.5
C8—C2—H2	108.6	C12—C11—C10	120.3 (2)
C3—C2—H2	108.6	C12—C11—H11	119.8
C1—C2—H2	108.6	C10—C11—H11	119.8
C4—C3—C2	113.96 (17)	C13—C12—C11	118.6 (2)
C4—C3—H3A	108.8	C13—C12—H12	120.7
C2—C3—H3A	108.8	C11—C12—H12	120.7
C4—C3—H3B	108.8	C12—C13—C14	121.8 (2)
C2—C3—H3B	108.8	C12—C13—Cl1	119.18 (17)
H3A—C3—H3B	107.7	C14—C13—Cl1	118.97 (16)
C5—C4—C3	112.78 (19)	C13—C14—C9	119.79 (19)
C5—C4—H4A	109.0	C13—C14—H14	120.1
C3—C4—H4A	109.0	C9—C14—H14	120.1
C5—C4—H4B	109.0	C16—C15—C20	118.3 (2)
C3—C4—H4B	109.0	C16—C15—C7	119.01 (19)
H4A—C4—H4B	107.8	C20—C15—C7	122.72 (18)
C4—C5—C6	114.49 (17)	C15—C16—C17	120.4 (2)
C4—C5—H5A	108.6	C15—C16—H16	119.8
C6—C5—H5A	108.6	C17—C16—H16	119.8
C4—C5—H5B	108.6	C18—C17—C16	120.8 (2)
C6—C5—H5B	108.6	C18—C17—H17	119.6
H5A—C5—H5B	107.6	C16—C17—H17	119.6
C8—C6—C5	107.32 (17)	C19—C18—C17	118.7 (2)
C8—C6—C7	106.25 (16)	C19—C18—H18	120.6
C5—C6—C7	116.39 (17)	C17—C18—H18	120.6
C8—C6—H6	108.9	C18—C19—C20	121.5 (2)
C5—C6—H6	108.9	C18—C19—Cl2	119.17 (19)
C7—C6—H6	108.9	C20—C19—Cl2	119.36 (18)
N1—C7—C15	111.44 (16)	C19—C20—C15	120.3 (2)
N1—C7—C6	109.14 (16)	C19—C20—H20	119.9
C15—C7—C6	112.37 (16)	C15—C20—H20	119.9
N1—C7—H7	107.9	C7—N1—C1	112.88 (15)
C15—C7—H7	107.9	C7—N1—H1A	108.0 (14)
C6—C7—H7	107.9	C1—N1—H1A	113.0 (14)
			
N1—C1—C2—C8	58.1 (2)	C1—C9—C10—C11	179.1 (2)
C9—C1—C2—C8	−177.77 (16)	C9—C10—C11—C12	0.2 (4)
N1—C1—C2—C3	−62.1 (2)	C10—C11—C12—C13	0.8 (4)
C9—C1—C2—C3	62.0 (2)	C11—C12—C13—C14	−1.3 (3)
C8—C2—C3—C4	−53.3 (2)	C11—C12—C13—Cl1	177.49 (19)
C1—C2—C3—C4	66.6 (3)	C12—C13—C14—C9	0.9 (3)
C2—C3—C4—C5	45.2 (3)	Cl1—C13—C14—C9	−177.89 (16)
C3—C4—C5—C6	−45.3 (3)	C10—C9—C14—C13	0.0 (3)
C4—C5—C6—C8	52.9 (2)	C1—C9—C14—C13	−179.61 (17)
C4—C5—C6—C7	−65.9 (2)	N1—C7—C15—C16	143.69 (19)
C8—C6—C7—N1	−58.4 (2)	C6—C7—C15—C16	−93.5 (2)
C5—C6—C7—N1	60.9 (2)	N1—C7—C15—C20	−37.3 (3)
C8—C6—C7—C15	177.42 (16)	C6—C7—C15—C20	85.5 (2)
C5—C6—C7—C15	−63.2 (2)	C20—C15—C16—C17	−1.1 (3)
C3—C2—C8—O1	−116.6 (2)	C7—C15—C16—C17	177.9 (2)
C1—C2—C8—O1	117.8 (2)	C15—C16—C17—C18	0.7 (4)
C3—C2—C8—C6	63.9 (2)	C16—C17—C18—C19	−0.3 (4)
C1—C2—C8—C6	−61.7 (2)	C17—C18—C19—C20	0.5 (4)
C5—C6—C8—O1	117.0 (2)	C17—C18—C19—Cl2	−179.20 (19)
C7—C6—C8—O1	−117.9 (2)	C18—C19—C20—C15	−1.0 (4)
C5—C6—C8—C2	−63.6 (2)	Cl2—C19—C20—C15	178.72 (16)
C7—C6—C8—C2	61.6 (2)	C16—C15—C20—C19	1.3 (3)
N1—C1—C9—C10	36.4 (3)	C7—C15—C20—C19	−177.8 (2)
C2—C1—C9—C10	−86.3 (2)	C15—C7—N1—C1	−174.09 (16)
N1—C1—C9—C14	−143.97 (18)	C6—C7—N1—C1	61.2 (2)
C2—C1—C9—C14	93.4 (2)	C9—C1—N1—C7	174.24 (16)
C14—C9—C10—C11	−0.6 (3)	C2—C1—N1—C7	−60.6 (2)

### Hydrogen-bond geometry (Å, °)

**Table:**

*D*—H···*A*	*D*—H	H···*A*	*D*···*A*	*D*—H···*A*
N1—H1A···O1^i^	0.83 (2)	2.35 (2)	3.129 (3)	155.4 (18)
C7—H7···O1^ii^	0.98	2.44	3.296 (2)	146

Symmetry codes: (i) *x*+1, *y*, *z*; (ii) *x*+1/2, −*y*+3/2, −*z*+2.
